# Time-dependent changes in monocyte subsets and gene expression patterns are associated with long-term recovery in patients with ischemic stroke

**DOI:** 10.21203/rs.3.rs-8980124/v1

**Published:** 2026-03-16

**Authors:** Juliane Tampé, Emanuela Monni, Yu-Ping Shen, Sara Palma-Tortosa, Emil Brogårdh, Arne Lindgren, Zaal Kokaia

**Affiliations:** Lund University; Lund University; National Defense Medical Center; Lund University; Lund University; Lund University; Lund University

**Keywords:** Ischemic stroke, Monocytes, Gene expression, Neuroinflammation, Neurological recovery, Immune trajectories

## Abstract

**Background:**

Post-stroke inflammation is increasingly recognized as a dynamic process that influences neurological recovery. However, how circulating monocyte subsets and their transcriptional programs evolve in relation to long-term functional outcomes in humans remains poorly defined. The objective of this study was to characterize the temporal dynamics of circulating monocyte subsets and their gene-expression profiles after ischemic stroke, and to identify immune signatures associated with neurological recovery, as assessed by longitudinal changes in the NIH Stroke Scale (NIHSS).

**Main text:**

Blood samples were collected from 37 patients with ischemic stroke at four time points: 24 hours, 3–5 days, 1 and 3 months after stroke, and from 37 age- and gender-matched control subjects. Monocyte subsets were quantified by flow cytometry. Gene expression profiling of isolated monocytes was performed using Fluidigm, with quantitative transcriptional analysis of immune-related genes. Longitudinal changes in monocyte subtype frequencies and gene expression were assessed, along with associations among immune parameters, sex, and neurological outcomes, to identify recovery-linked immune trajectories.

Total circulating monocyte levels increased during the acute and early subacute phases, with outcome- and sex-dependent differences. Classical monocytes declined at 3 months, whereas intermediate monocytes increased at 3–5 days in patients with favorable recovery.

Gene expression analyses showed early attenuation of inflammatory and costimulatory signaling. Low TSPO expression at 24 hours was associated with greater neurological deficit, as indicated by higher NIHSS, and reduced CD86 and IL-1β expression at 3–5 days was observed in patients with more severe neurological impairment. At 3 months, increased CCR2 expression suggested ongoing monocyte recruitment and persistent immune activity. Increased expression of CD91, CD36, and TGM2 was associated with favorable outcomes, whereas higher expression of CD11c, CCR2, and CX3CR1 was associated with a poorer prognosis. CD91 emerged as a marker associated with greater longitudinal improvement in NIHSS, and CD36 expression in intermediate monocytes revealed a previously unrecognized immune signature linked to recovery in human stroke.

**Conclusions:**

These findings demonstrate that stroke recovery is associated with coordinated, time-dependent reprogramming of circulating monocytes rather than persistent inflammatory activation. Identification of monocyte-based transcriptional signatures associated with functional outcomes supports the potential of immune profiling to improve prognostic stratification and inform future immune-targeted strategies for stroke recovery.

## Introduction

Ischemic stroke remains a leading cause of death and long-term disability worldwide, often resulting in persistent neurological impairment and long-term disability(1). Although advances in acute reperfusion therapies, including thrombolysis and mechanical thrombectomy, have improved outcomes for selected patients, these benefits are limited to a narrow therapeutic window and a minority of individuals (2). Consequently, most stroke survivors experience residual neurological deficits, underscoring the need to better understand the biological processes that support recovery beyond the acute phase (3).

Neuroinflammation is a central component of the pathophysiological response to ischemic injury and exerts context-dependent effects on both tissue damage and repair. Microglia, the brain’s resident immune cells, are rapidly activated after stroke and initiate inflammatory cascades that influence neuronal injury, synaptic remodeling, and vascular integrity (4, 5). In parallel, ischemic stroke induces a systemic immune response characterized by the mobilization of peripheral innate immune cells that interact dynamically with the injured brain. Among these, circulating monocytes are a heterogeneous population that can contribute to both inflammatory injury and tissue repair, depending on their activation state and local microenvironment (6, 7).

Human monocytes are commonly classified into three subsets based on surface marker expression: classical (CD14^+^/CD16^−^), intermediate (CD14^+^/CD16^+^), and non-classical (CD14^−^/CD16^+^) (8). Classical monocytes are produced in the bone marrow and released into the circulation, where they persist for approximately 1–2 days (9, 10). These cells exhibit strong phagocytic and antigen-presenting capacities and are the primary responders to inflammatory stimuli. In the absence of tissue recruitment, classical monocytes undergo stepwise differentiation into intermediate monocytes, which display enhanced inflammatory and immunoregulatory functions, and subsequently into non-classical monocytes, which are involved in endothelial surveillance and immune homeostasis (10, 11). This maturation process is critically dependent on chemokine signaling, including CCR2 receptor expression (12).

Experimental studies have shown that monocyte-derived immune responses play an important role in shaping post-stroke inflammation and repair. Our previous work in animal models demonstrated that monocyte-driven immune modulation promotes anti-inflammatory pathways that support neurovascular remodeling and functional recovery (13). In addition, transplantation of anti-inflammatory-primed monocyte-derived macrophages into the ischemic brain improved motor and cognitive outcomes in mice (14). In humans, altered distributions of monocyte subsets and imbalances between inflammatory and reparative phenotypes have been linked to stroke severity, outcome, and post-stroke complications, including infections and immune suppression (15). However, the temporal evolution of these monocyte responses and their relationship to long-term neurological recovery remain incompletely understood.

Age and biological sex are important modifiers of immune responses and may influence monocyte-mediated recovery after stroke. Aging is associated with chronic low-grade inflammation, impaired immune regulation, and altered monocyte function (16, 17). Age-related changes in monocyte number and function may limit the capacity to modulate neuroinflammation and support tissue repair following ischemic injury (18). In addition, sex-specific differences in monocyte phenotypes and inflammatory responses have been reported across multiple conditions (19, 20), yet their impact on post-stroke monocyte dynamics and recovery remains underexplored. We recently demonstrated that age and sex significantly influence monocyte subset distributions and expression of inflammation- and regeneration-associated genes in healthy individuals, with female monocytes showing more pronounced age-related alterations (21).

Despite growing interest in immune-mediated mechanisms of stroke recovery, relatively few studies have longitudinally characterized circulating monocyte subsets and their gene expression profiles in relation to clinical outcomes (15, 22, 23). In particular, time-resolved analyses linking early immune responses to long-term neurological recovery in humans are lacking. To address this gap, we conducted a longitudinal study of ischemic stroke patients, analyzing circulating monocyte subsets and their neuroinflammatory and regeneration-associated gene expression profiles at acute (24 hours), subacute (3–5 days), and chronic (3 months) phases of ischemic stroke, and relating these profiles to neurological impairment assessed by the National Institutes of Health Stroke Scale NIHSS (24).

In this study, we identify distinct time-dependent patterns in monocyte subset composition and transcriptional programs that are associated with neurological recovery and influenced by biological sex. We further identify recovery-associated gene expression signatures in classical, intermediate, and non-classical monocytes during the acute and subacute phases that relate to neurological outcome at 3 months. Specifically, increased expression of genes linked to phagocytic and inflammation-resolving functions, including CD91, CD36, and TGM2, was associated with favorable recovery, whereas elevated expression of inflammatory markers such as CD11c, CCR2, and CX3CR1 was associated with poorer outcomes.

Together, these findings highlight the dynamic and heterogeneous nature of monocyte responses after ischemic stroke and support the idea that longitudinal immune profiling can reveal biologically meaningful immune trajectories associated with recovery. Such insights may improve prognostic stratification and inform future hypothesis-driven studies aimed at mitigating the detrimental effects of neuroimmune mechanisms and promoting repair in ischemic stroke.

## Material and methods

### Standard protocol approvals, registrations, and patient consents

All study procedures adhered to the ethical guidelines set by the Regional Ethics Review Board in Lund, Sweden. The study protocol was approved as part of the Lund Stroke Register project (ethical permits diary numbers 2016/179; approved on 4.8.2016, 2017/357; approved on 2.5.2017, and 2017/879 approved on 13.11.2017) and complied with the principles outlined in the Declaration of Helsinki. Written informed consent was obtained from all participants or, where applicable, from their next of kin, for blood sampling, genetic and protein analyses, and clinical evaluations. Before enrollment, participants were provided with comprehensive information about the study procedures, including their right to withdraw at any time without consequence. Any previously collected samples were destroyed if the participant was later excluded from the study. The ethical permit approvals covered venous blood sampling, magnetic resonance imaging (MRI) scans within the first three months post-stroke, and various non-invasive neurological assessments. This study does not include any experiments performed on live vertebrates and/or higher invertebrates. The study does not report on a clinical trial, and it does not disclose any recognizable persons in photographs, videos, or other personal information.

The inclusion criteria for the study were as follows: patients aged 20 to 85 years (inclusive), of either sex, with a first-ever acute ischemic stroke confirmed by computed tomography (CT) or MRI, and an NIHSS score of 1 or higher. Patients who had received reperfusion therapy were also eligible for inclusion. Exclusion criteria included pregnancy, systemic inflammatory conditions (e.g., rheumatoid arthritis, systemic lupus erythematosus), and hematologic disorders affecting the immune function.

To ensure balanced and representative control samples, we selected age- and sex-matched blood samples from volunteer control donors in the Lund Stroke Register (LSR) who had no history of stroke at the time of donation. We paired each patient with a donor of approximately the same age (± 4 years) and, when possible, of the same sex, to achieve statistically comparable average ages across all groups.

All samples were pseudonymized and coded to prevent personal identification. To minimize bias, samples were randomized, and both data collection and analysis were conducted in a blinded manner.

### Neurological tests

Patients enrolled in this study, conducted in collaboration with the Lund Stroke Register of Skåne University Hospital, had neurological status assessed longitudinally using the NIHSS at baseline (within 24 hours), 3–5 days, 1 month, 3 months, and 1 year after stroke. The NIHSS is a validated tool that quantifies stroke severity based on neurological deficits and assesses key neurological functions, including consciousness, visual fields, motor strength, sensation, language, and attention. It enables standardized comparisons of stroke impact and progression over time. Because the NIHSS quantifies neurological deficit severity rather than global functional independence, the term “*neurological recovery”* in this study refers specifically to changes in NIHSS over time and does not imply functional, stroke recovery (25) as measured by scales such as the modified Rankin Scale (mRS).

Long-term neurological outcome was defined as the absolute NIHSS score at 3 months and 1 year. Neurological recovery was defined as the change in NIHSS from baseline (within 24 hours) to follow-up. For categorical analyses, patients were classified based on their longitudinal change in NIHSS. Patients showing a decrease of 1 or more NIHSS points from baseline to the chronic phase (3 months and/or 1 year) were categorized as exhibiting neurological improvement (Recovery), whereas those with unchanged or increased NIHSS were categorized as having no neurological improvement (No Recovery; see Supplementary Table 1). This approach distinguishes between residual neurological impairment (outcome) and the magnitude of neurological improvement (Recovery).

### Blood sample and peripheral blood mononuclear cell collection

Research nurses at the Department of Neurology, Skåne University Hospital, collected 10 mL of venous blood from 74 participants (37 stroke patients and 37 healthy donors; see Supplementary Table 1) using heparin-treated, EDTA-coated blood collection tubes (Vacutainers, BD, Sweden). Samples were kept at room temperature (RT) and processed within 24 hours. Peripheral blood mononuclear cells (PBMCs) were isolated with SepMate tubes (StemCell Technologies, Sweden) and Lymphoprep (Serumwerk, Germany), following the manufacturer’s instructions. All procedures were performed in a biosafety level 2 laboratory under a laminar flow hood, using only animal-free reagents to minimize monocyte activation. Isolated PBMCs were cryopreserved in Stem-CellBanker (Amsbio, UK) at −80°C, then transferred to either a −150°C or liquid nitrogen freezer (−180°C) for long-term storage.

For subsequent analyses, participants were stratified by clinical characteristics and study objectives. Specifically, flow cytometry analysis included all 74 participants, whereas gene expression analyses were performed in a subset of 30 donors (15 stroke patients and 15 controls). Further subgrouping was based on sex (male, female), stroke severity Further subgrouping was based on sex (male, female), stroke severity (“mild stroke”, NIHSS 1–5 and “moderate-to-severe stroke” NIHSS, 6–20).

### Flow cytometry analysis and sorting

Frozen PBMCs were thawed at RT, washed with Dulbecco’s Phosphate-Buffered Saline (DPBS) containing 2% recombinant-human serum albumin (rHSA), resuspended in antibody-containing buffer, and incubated at 4°C for 30–60 minutes. After incubation, cells were washed, pelleted, and filtered for flow cytometry analysis. Consistent with our previous work (21), primary human monocytes and their subtypes were identified by CD91 surface expression and differential CD14 and CD16 expression. Although used for monocyte identification, CD91 was also included in the gene expression analyses as it encodes a receptor with established roles in innate-adaptive immune regulation. B-, NK-, and T-cells were distinguished by expression of CD19, CD56, and CD3, respectively. Antibodies included CD91-PE, CD14-APC, CD16-BV421, CD3-PE-Cy7, CD19-BB515, and CD56-BV605 (BD Biosciences, Sweden), with non-viable cells excluded by DRAQ7 staining (Supplementary Table 2).

For monocytes and each of the three subtypes, biological duplicates of 20 cells were sorted on the Aria^™^ II FACS system (BD Biosciences, Sweden), as described by Tampé et al. (21). Baseline autofluorescence was measured using unstained cells, and single-stained controls were used to correct for partial spectral overlap. Gating strategies used fluorescence minus one (FMO) controls and were confirmed by re-analysis and backgating.

### Gene expression analysis using Fluidigm (multiplexed RT-qPCR)

A chip-based high-throughput RT-qPCR platform (Fluidigm) was used to examine gene expression in monocytes and their subtypes, analyzing 40 genes associated with brain inflammation and regeneration (Supplementary Table 3). Regenerative genes (n=36) were selected from a comprehensive literature review, focusing on those expressed by monocytes/macrophages involved in neuroinflammation and stroke recovery. The panel also included negative and technical controls to ensure adequate quality and reliability. RT-qPCR data from each Fluidigm chip were analyzed using the BioMark HD software with a quality threshold of 0.65, linear baseline correction, and automatic global cycle threshold (CT) (21).

Data were compiled using the software R (https://github.com/JTampe/Monocytes). Non-detected runs were assigned a CT value of 35 (26). Missing data were imputed using Gene, Subtype, Category, Sex, and Age (27). Normalization used the median gene expression within each sample to obtain relative expression (dCT) (28).

### Statistical analyses

Demographic statistics, including sample size (N), age, and sex distributions, were computed for both the control and patient groups. Age distributions, both overall and split by biological sex, stroke category, or recovery status, were compared using an unpaired t-test or Wilcoxon rank-sum test, and sex distribution was compared using a chi-square test.

For general and subtype monocyte frequencies, one million events per sample were collected using DIVA software on the FACSAria^™^II cell sorter. The Wilcoxon rank-sum test was used to compare patients and control subjects at each time point after stroke. Results are presented as bar plots showing mean cell-type percentages with standard errors of the mean (SEM). This analysis was performed for all monocyte frequencies across all patients and control subjects, with additional stratification by biological sex and recovery status.

The dCT values were normalized by z-scoring: the median gene expression within the sample was set to 0, and expression values were scaled based on their standard deviation. To minimize the influence of outliers, z-scores were capped at ±3, corresponding to values within three standard deviations of the median. Technical and biological replicates were then combined by calculating the mean z-scored expression values for statistical analysis. A positive z-score indicates that a gene was upregulated relative to the sample median, while a negative z-score indicates downregulation.

NIHSS-related changes in gene expression and cell type frequencies were assessed using linear regression (Supplementary Tables 4 and 5). Outliers were defined as values more than three standard deviations from the mean. Results included the Pearson correlation coefficient (r), slope, and intercept, along with corresponding 95% confidence intervals. Both flow cytometry data (cell type frequencies) and gene expression data were confirmed to be normally distributed. A P-value < 0.05 was considered statistically significant.

### Graphics and Design

**Figure 1:**
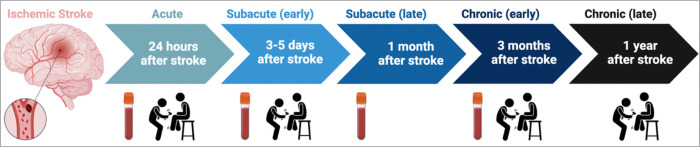
Workflow of the presented study. After ischemic stroke and consent to join the study, patients undergo neurological testing (e.g., NIHSS), and 10 mL of blood is collected. Neurological testing is performed within 24 hours, 3–5 days, 3 months, and 1 year after stroke. Blood samples for flow cytometry and gene expression analysis are collected within 24 hours, 3–5 days, 1 month, and 3 months after stroke.

**Figure 2:**
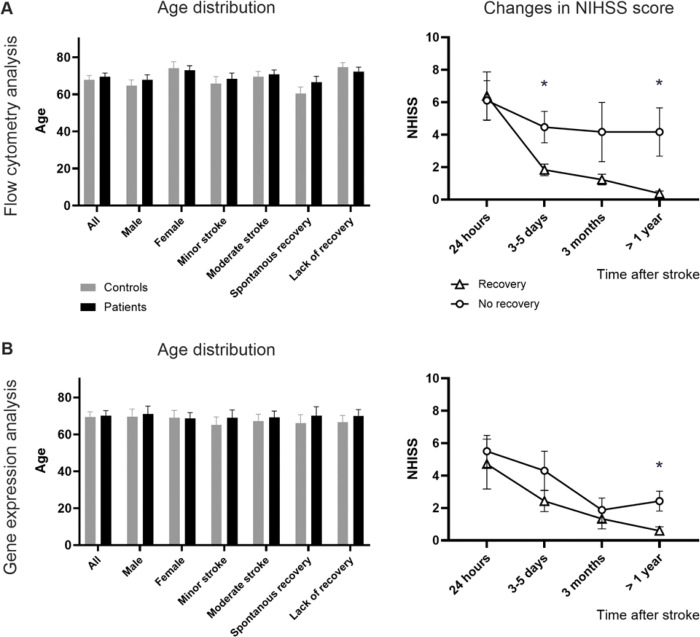
Patient characteristics used to assess neurological deficits, including age distribution and NIHSS score. The age distribution of donors (N=37 each) included in the flow cytometry analysis (A) and their NIHSS score for the Recovery group (B), and donors included in the gene expression assessment (rt-qPCR/Fluidigm, N=15 each) are shown with their age (C) and NIHSS score (D). Mild strokes are defined by an NIHSS score of 1–5, and mild to moderate strokes by an NIHSS score of 5–14. NIHSS scores are assessed at acute (0–24 hours), subacute (3–5 days), and chronic (3 months and >1 year) time points after stroke. Control subjects are shown in gray, patients in black. Patients are grouped by Recovery (triangle) and No recovery (circle). Data are presented as mean ± SEM, compared using an unpaired t-test, and generated using PRISM software.

**Figure 3:**
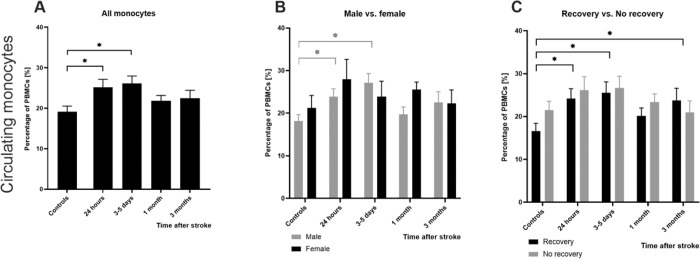
Temporal changes in peripheral monocytes after ischemic stroke. (A) The proportion of all circulating monocytes within isolated, viable PBMCs is compared with age- and sex-matched control subjects at acute (0–24 hours), subacute (3–5 days), and chronic (3 months) time points after stroke (N=37). (B) All monocytes from controls are compared, split by biological sex (N(male)=25; N(female)=12). (C) All monocytes from control subjects are compared, split by recovery status, defined by NIHSS score in the chronic phase (N(Recovery)=18; N(No recovery)=19). Peripheral monocyte frequencies are presented as a percentage of all viable PBMCs. Data are shown as mean ± SEM, compared using multiple unpaired t-tests (Wilcoxon), and generated using PRISM software.

**Figure 4:**
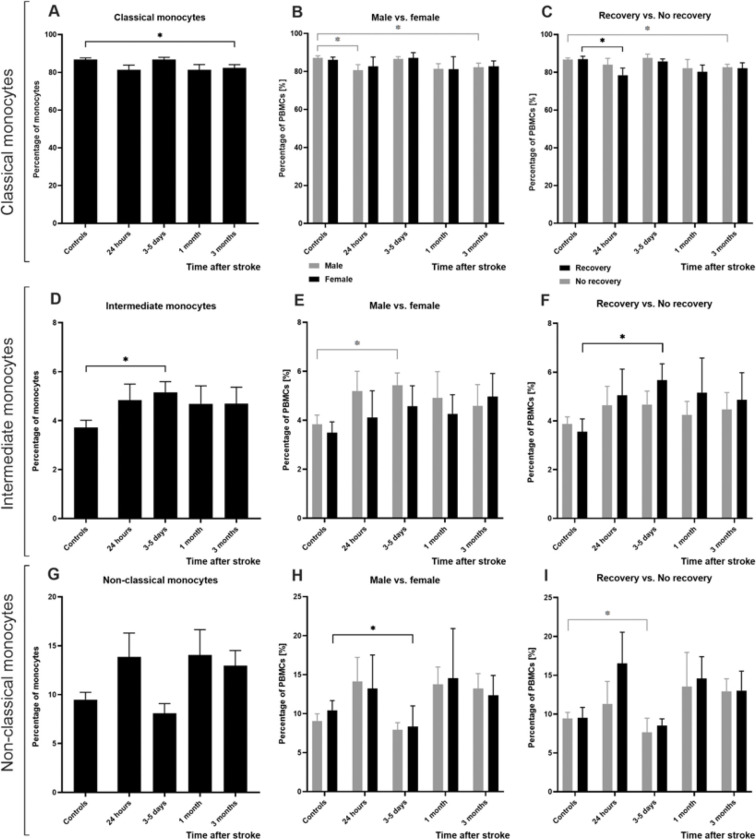
Temporal changes in monocyte subtypes after ischemic stroke. The proportions of (A) classical, (D) intermediate, and (G) non-classical monocytes among all peripheral monocytes are compared with age- and sex-matched controls at acute (0–24 hours), subacute (3–5 days), and chronic (3 months) time points after stroke (N=37). (B) Classical, (E) intermediate, and (H) non-classical monocytes from controls are compared with those from patients, split by biological sex (N(male)=25; N(female)=12). (C) Classical, (F) intermediate, and non-classical monocytes from controls are compared with those from patients, split by recovery status, defined by NIHSS score in the chronic phase ((Recovery) N =18; (No recovery) N=19). Monocyte subtype frequencies are presented as a percentage of all viable PBMCs. Data are shown as mean ± SEM, compared using the unpaired Wilcoxon test, and generated using PRISM software.

Figure 1 was created in BioRender (https://BioRender.com/mm8lopy Tampe, J. (2025)). Figures 2, 3, and 4 were generated using PRISM (Version 10, GraphPad). All other figures and tables were produced using R software (R Core Team, 2021). R is a language and environment for statistical computing (R Foundation for Statistical Computing, Vienna, Austria. URL: https://www.R-project.org/).

## Results

### Study design and patient characteristics

To investigate the immune response after ischemic stroke, we assessed neurological deficits and collected blood samples from patients at multiple time points (Figure 1). In total, we collected blood and performed neurological assessments for 37 patients, 19 of whom had experienced a mild stroke and 18 a moderate or severe stroke. The patient sample had a median age of 69.5 ± 12.2 years, with 12 females (32%) and 25 males. The control subjects had a median age of 67.8 ± 14.8 years, with 17 females (40%) and 26 males. The age distribution between patients and control subjects (P = 0.576) and other group comparisons (Figure 2A and C; Supplementary Table 6) were not significant.

Patients with a decreased NIHSS score in the chronic stage after stroke (at 3 months and 1 year) were categorized as recovering well (“Recovery”), and patients with a persistently higher or unchanged NIHSS score were classified as “No recovery” (Figure 2B and 2D; Supplementary Table 7). The categorization was determined by a medical professional at the LSR, who compiled patients’ NIHSS scores across all time points and compared them to determine whether the score decreased, increased, or remained unchanged. Particular attention was paid to the first and last time points (within 24 hours and after 1 year, respectively). Patients classified in the No recovery group and included in the flow cytometry analysis exhibited significantly higher NIHSS scores at 3–5 days and 1 year after stroke. In the gene expression analysis, No recovery patients showed elevated NIHSS scores only at 1 year post-stroke.

In this exploratory study, flow cytometry data were collected from all 37 patients and 37 matched control subjects, and gene expression analysis using Fluidigm was performed on a subset of 15 patients and 15 healthy donors.

### The relative numbers of monocytes increase shortly after a stroke

Circulating monocytes contribute to post-stroke inflammation and recovery. We performed flow cytometry analysis on PBMCs to assess their dynamics and quantify monocyte proportions while excluding B-, NK-, and T-cells using the markers CD19, CD56, and CD3, respectively. Consistent with our previous work (21), pan-monocytes were identified based on the surface marker CD91(29). We analyzed these proportions over time and compared them between stroke patients and controls, as well as between biological sexes and recovery groups (Figure 3; Supplementary Table 8).

We found that the proportion of circulating monocytes was significantly increased within 24 hours and at 3–5 days after stroke (at 24 hours, p= 0.031; at 3–5 days, p = 0.003; Figure 3A). However, when analyzed separately by sex, these temporal changes were observed only in male patients (at 24 hours, p = 0.045; at 3–5 days, p = 0.001; Figure 3B). Further analysis confirmed this pattern (at 24 hours, p = 0.022; at 3–5 days, p = 0.008) and additionally revealed an increase in monocyte percentage at 3 months after stroke in patients who recovered (at 3 months, p = 0.023; Figure 3C).

### Dynamic changes in monocyte subtypes after stroke are linked to sex and recovery

Human monocytes are classified into classical, intermediate, and non-classical subtypes based on CD14 and CD16 expression. Each subtype has distinct roles in immune regulation and repair. We analyzed temporal changes in these subtypes among stroke patients and their relationships with biological sex and recovery status (Figure 4; Supplementary Table 8).

When examining classical monocyte subtype distributions, we observed a significant decrease at 3 months after stroke compared with the control group (at 3 months, p = 0.041; Figure 4A), a pattern also observed in males (at 3 months, p = 0.038; Figure 4B) and in the No recovery group (at 3 months, p = 0.035; Figure 4C). Furthermore, classical monocytes significantly decreased within 24 hours post-stroke in male patients (within 24 hours, p = 0.048; Figure 4B) and in the recovery group (within 24 hours, p = 0.017; Figure 4C).

For intermediate monocytes, we revealed a significant increase at 3–5 days after stroke (at 3–5 days, p = 0.006; Figure 4D), which was also observed in both the male group (at 3–5 days, p = 0.007; Figure 4E) and the recovery group (at 3–5 days, p = 0.010; Figure 4F).

Analysis of non-classical monocyte proportions showed dynamic changes but no significant difference compared with control subjects (Figure 4G). However, subgroup analysis revealed a significant reduction in non-classical monocytes among female patients (at 3–5 days, p = 0.023; Figure 4H) and among patients in the no-recovery group (at 3–5 days, p = 0.009; Figure 4I).

### Inflammation-related genes are differentially expressed in monocyte subtypes after stroke

We investigated the relationship between neuroinflammatory and regeneration-associated gene expression in circulating monocytes and NIHSS scores at multiple time points after stroke. Because higher NIHSS scores relative to baseline indicate greater neurological impairment, and lower scores reflect recovery, we analyzed key gene-expression patterns to identify acute, subacute, and chronic shifts in immune-response dynamics and their potential impact on neurological outcomes (Table 1).

Across all monocytes, TSPO gene expression (p = 0.004, r = −0.696) within 24 hours was strongly negatively correlated with NIHSS. At 3–5 days, the expression of the pro-inflammatory genes CD86 (p = 0.010, r = −0.642) and IL-1β (p = 0.018, r = −0.601) was also negatively correlated with NIHSS scores, indicating a moderately strong association with improved neurological status relative to baseline. At 3 months post-stroke, CCR2 (p = 0.002, r = 0.777) and STAT6 (p = 0.021, r = 0.629) gene expressions were positively correlated with NIHSS, suggesting persistent inflammatory activity in individuals with greater neurological deficits. In contrast, SLC24A4 gene expression (p = 0.049, r = −0.554) was negatively correlated with NIHSS score (Supplementary Table 5).

Understanding how different monocyte subtypes contribute to neurological recovery after stroke can provide insights into their distinct roles in neuroinflammation and repair. In classical monocytes, TSPO gene expression (p = 0.003, r = −0.708) was significantly negatively correlated with NIHSS at 24 hours, consistent with the trend observed across all monocytes. At 3 months, CCR2 gene expression (p = 0.010, r = 0.686) was positively correlated with NIHSS, whereas SLC24A4 (p = 0.044, r = −0.566) and CD91 gene expression, an important immune regulator (p = 0.049, r = −0.555), were negatively correlated with NIHSS, consistent with transcriptional shifts potentially related to recovery.

In intermediate monocytes, downregulation of CD33 expression (p = 0.027, r = −0.567) at 3–5 days post-stroke, and upregulation of CCR2 (p = 0.049, r = 0.556) and CD11b (p = 0.022, r = 0.627) at 3 months were correlated with No recovery of NIHSS relative to baseline values, indicating subtype-specific transcriptional changes over time.

In non-classical monocytes, the pro-inflammatory gene TLR8 (P = 0.032, r = 0.574) was positively correlated with NIHSS, suggesting early inflammatory activation in patients with worse neurological status. At 3–5 days after stroke, the pro-inflammatory gene CD40 was significantly downregulated (P = 0.022, r = −0.606), with lower expression in patients with higher NIHSS scores. Similarly, at 3 months, CD91 (P = 0.031, r = −0.599), IL-10 (P = 0.035, r = −0.587), and SLC24A4 (P = 0.006, r = −0.714) were downregulated, each moderately to strongly negatively correlated with NIHSS. This suggests potential links between these pathways and favorable long-term recovery at 3 months.

### Inflammation-related genes are associated with longitudinal neurological improvement after stroke

**Figure 5:**
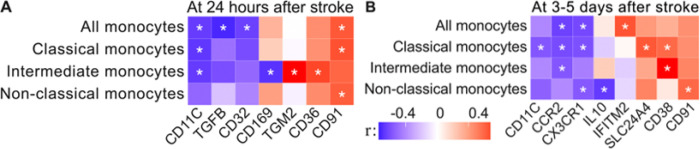
Gene expression correlating with recovery at 3 months. Heatmap of predictive gene expression for the NIHSS score at 3 months. The strength of the association of (A) acute (0–24 hours) and (B) subacute (3–5 days) gene expression with relative recovery at 3 months post-stroke is shown by the Pearson correlation coefficient (r), ranging from −1 (perfect negative correlation, blue) to 0 (no correlation) to 1 (perfect positive correlation, red). Relative recovery is calculated as the change in NIHSS scores from stroke onset to 3 months post-stroke, normalized to the NIHSS score at stroke onset (0 indicates no recovery, and 1 indicates full recovery). Significant genes, defined by a P-value below 0.05, are marked with an asterisk (*).

We performed gene expression analysis at acute and subacute time points to identify molecular signatures associated with post-stroke recovery. We then correlated these expression patterns with neurological recovery at 3 months to identify markers associated with neurological recovery after stroke (Figure 5; Supplementary Table 9).

At the acute stage (within 24 hours after stroke), the correlation between gene expression and long-term NIHSS scores revealed several significant associations: downregulation of the monocyte adhesion gene CD11c was linked to better long-term recovery in all (P = 0.033), classical (P = 0.028), and intermediate monocytes (P = 0.033). Decreased expression of the anti-inflammatory TGFβ (P = 0.007) and immune modulator CD32 (P = 0.037) was associated with recovery in all monocytes. Reduction in the pro-inflammatory gene CD169 in intermediate monocytes (P = 0.024) was associated with a positive recovery trajectory. In contrast, upregulation of the macrophage activator TGM2 (P = 0.007) and pro-inflammatory gene CD36 (P = 0.024) in intermediate monocytes was associated with recovery. Increased expression of the cytokine modulator CD91 in all (P = 0.041), classical (P = 0.32), and non-classical monocytes (P = 0.047) was also correlated with recovery (Figure 5A).

At the subacute stage (3–5 days after stroke), several additional gene expression patterns were linked to recovery. Downregulation of the monocyte adhesion molecule CD11c in classical monocytes (P = 0.036) was correlated with better recovery, as was lower expression of the monocyte infiltration gene CCR2 in all (P = 0.016), classical (P = 0.024), and intermediate monocytes (P = 0.036). The anti-inflammatory gene CX3CR1 was also downregulated in all (P = 0.022), classical (P = 0.029), and non-classical monocytes (P = 0.041), suggesting a potential role in recovery. IL-10, another anti-inflammatory mediator, was reduced in non-classical monocytes (P = 0.016) and associated with better recovery, suggesting a complex regulation of inflammation. In contrast, upregulated expression of the gene IFITM2, involved in interferon response and antiviral immunity, was linked to improved recovery in all monocytes (P = 0.013), as was the cellular calcium ion homeostasis regulator SLC24A4 in classical monocytes (P = 0.017). Additionally, the immune modulator CD38 was associated with recovery in classical (P = 0.032) and intermediate monocytes (P = 0.001). Finally, increased CD91 expression in non-classical monocytes (P = 0.031) was associated with better recovery, further highlighting the role of immune modulation in post-stroke repair.

## Discussion

Circulating monocytes are mobilized after acute ischemic stroke and contribute to recovery through dynamic, time-dependent changes in their abundance and transcriptional programs (13, 30, 31). This exploratory longitudinal study extends prior work by linking peripheral immune dynamics to long-term neurological outcome in a well-characterized human cohort. By integrating repeated immune profiling with neurological outcomes, we identified distinct temporal and subtype-specific monocyte patterns associated with biological sex and recovery status, highlighting that post-stroke immune responses evolve over time rather than representing static inflammatory states.

Flow cytometric analyses demonstrated a significant increase in circulating monocytes within the first 24 hours and at 3–5 days after stroke, consistent with previous reports (15). This early expansion was most pronounced in male patients and in individuals who subsequently achieved greater neurological improvement. An additional increase in monocyte levels at 3 months was observed specifically in the recovery group, suggesting that sustained peripheral immune activity may accompany longer-term repair processes. Consistent with prior observations, the early predominance of classical monocytes was associated with an unfavorable outcome, whereas higher proportions of intermediate and non-classical monocytes were associated with recovery (15), underscoring the importance of monocyte subset composition rather than absolute cell counts alone.

Subtype-specific analyses further indicated that classical monocytes, which support inflammatory responses (32), declined significantly at 3 months after stroke, particularly in male patients and those without recovery. Notably, classical monocytes also decreased as early as 24 hours in patients who subsequently recovered, suggesting that early attenuation of classical monocyte-driven inflammation may be beneficial. In contrast, intermediate monocytes increased significantly at 3–5 days in male patients and those with favorable recovery, consistent with their established roles in immunomodulation, phagocytosis, and tissue repair (15, 32). Non-classical monocytes did not show overall changes but declined in female patients and those without recovery, potentially reflecting impaired vascular surveillance and immune homeostasis, consistent with previous reports (33).

In line with previous studies, classical monocytes declined at 3 months, particularly among male patients and those with poor recovery (31). While classical monocytes are critical for early immune activation and cytokine production, sustained predominance may promote chronic inflammation and impair recovery (30, 34). Intermediate monocytes increased at 3–5 days in patients with better recovery, supporting a role in inflammatory modulation and neurovascular remodeling (31). Although non-classical monocytes did not change globally, reductions in females and in patients without recovery suggest compromised tissue surveillance and immune homeostasis in these subgroups (33).

Together, these findings support a model in which monocyte responses after stroke follow distinct immune trajectories rather than a uniform inflammatory course. In this study, immune trajectories refer to the coordinated, time-dependent changes in circulating monocyte subset composition and transcriptional programs that evolve after ischemic stroke and are associated with long-term neurological outcomes. Early phases appear to be dominated by inflammatory activation, whereas later stages in patients with greater reductions in NIHSS scores are characterized by a transition toward reparative and inflammation-resolving immune programs. Such trajectory-based framing is particularly relevant to longitudinal human studies, in which repeated measurements provide insight into immune adaptation over time.

Sex-specific immune differences were evident and may contribute to heterogeneity in recovery. Higher acute monocyte levels in males have previously been linked to improved outcomes (33). In our cohort, males exhibited greater reductions in classical monocytes at 24 hours and 3 months, potentially reflecting androgen-related immune modulation (19). In contrast, females demonstrated relatively greater recruitment of intermediate and non-classical monocytes, which may support the resolution of inflammation and tissue repair (20). Although the study was not powered for definitive sex-stratified analyses, these findings align with growing evidence that sex influences post-stroke immune responses and recovery (20) and should be considered hypothesis-generating.

Gene expression profiling provided additional mechanistic insight into recovery-associated immune trajectories. Early downregulation of TSPO at 24 hours correlated with worse neurological impairment, suggesting that preserved TSPO expression may reflect metabolic or reparative immune activity during the acute phase. TSPO encodes the 18-kDa translocator protein, a mitochondrial marker upregulated in microglia and monocyte-derived cells during inflammatory responses (35), and is widely used in positron emission tomography (PET) imaging to monitor neuroinflammation (36).

Downregulation of CD86 and IL-1β at 3–5 days was associated with increased NIHSS, highlighting the complex timing-dependent relationship between immune activation and recovery (14, 37). At 3 months, increased CCR2 expression correlated with worse outcome, consistent with chronic inflammatory recruitment observed in experimental stroke models (38), although CCR2 + monocytes may exert context-dependent reparative functions during earlier stages (39).

Downregulation of SLC24A4 and CD91 was associated with improved neurological outcomes, suggesting involvement of calcium-regulatory and immune-signaling pathways relevant to cellular stress resolution (37, 40). IL-10 expression was inversely correlated with NIHSS, consistent with its established anti-inflammatory and neuroprotective roles during later stages of recovery (34). Collectively, these transcriptional changes support the idea that recovery is associated with the attenuation of sustained inflammatory signaling and the engagement of immune programs linked to resolution and repair.

Because a time-resolved monocyte immunophenotypic signature associated with long-term recovery has not been established, we performed exploratory correlation analyses at 24 hours and 3–5 days. Early downregulation of CD11c, CD32, TGF, and CD169, genes involved in monocyte activation, phagocytosis, and inflammation (41–44) was associated with recovery, suggesting that early attenuation of monocyte activation may be protective. In contrast, upregulation of TGM2, CD36, and CD91, particularly within intermediate monocytes, was associated with favorable outcomes. TGM2 has been implicated in ischemia-induced injury in murine models (45). Notably, this study provides the first evidence that increased CD36 expression in intermediate monocytes at 24 hours is associated with improved functional recovery in human stroke, consistent with preclinical studies demonstrating CD36-mediated efferocytosis and neuroprotection (46). CD91 remained consistently associated with greater improvement in NIHSS across monocyte subsets and time points, supporting its potential relevance as a stable immune marker.

At 3–5 days, reduced expression of CCR2, CX3CR1, CD11c, and IL-10 across specific subsets, and increased expression of IFITM2, SLC24A4, CD38, and CD91 were associated with better recovery. Emerging evidence suggests roles for SLC24A4 and CD169 in monocyte trafficking and activation (47, 48). CD38 exhibits context-dependent neuroimmune effects, with roles in both injury and repair depending on timing (49). To date, no clinical studies have linked CD38 expression in intermediate monocytes to recovery, making this observation novel and hypothesis-generating.

Several limitations should be considered. The cohort size was modest, reflecting the logistical constraints of longitudinal immune profiling in human stroke, and limits subgroup analyses, including sex stratification. Gene expression profiling was targeted rather than transcriptome-wide, prioritizing biologically interpretable pathways but potentially overlooking additional mechanisms. Immune profiling was limited to peripheral blood and may not fully capture immune dynamics in the central nervous system. However, circulating monocytes are the principal source of infiltrating macrophages after stroke (50) and can be collected and examined via a minimally invasive procedure. Functional assays were not performed to establish causality. However, the analyses were exploratory and correlation-based. Follow-up studies incorporating additional functional and cognitive outcome measures may further refine the relationships between immune markers and outcomes. Despite these limitations, the repeated-measures longitudinal design and integration of immune and clinical data provide unique insight into dynamic monocyte immune trajectories associated with greater improvement in NIHSS.

In conclusion, this study demonstrates that neurological recovery, as assessed by longitudinal change in NIHSS, is associated with time-dependent reprogramming of circulating monocytes rather than with persistent inflammatory activation. Our findings suggest that long-term recovery after ischemic stroke may be influenced by a dynamic “immune trajectory” of circulating monocytes. We propose that favorable recovery is linked to the subacute expansion of intermediate monocytes and the upregulation of tissue-clearing genes (*CD36*, *TGM2*, *CD91*) that facilitate tissue repair. Moreover, our data suggest that poor outcomes may be associated with a failure to resolve systemic inflammation, characterized by sustained *CCR2* expression and a deficit in non-classical monocytes, which are essential for vascular surveillance. Identifying recovery-associated monocyte trajectories and transcriptional programs provides mechanistic insight into peripheral immune contributions to neuroinflammation and repair and supports the potential value of longitudinal immune profiling for prognostic stratification and future hypothesis-driven neuroimmune research.

## Supplementary Material

Supplementary Files

This is a list of supplementary files associated with this preprint. Click to download.

• SupplementaryTable5.pdf

• SupplementaryTable2.pdf

• SupplementaryTable1.pdf

• SupplementaryTable6.pdf

• SupplementaryTable3.pdf

• SupplementaryTable4.pdf

• SupplementaryTable7.pdf

• SupplementaryTable8.pdf

• SupplementaryTable9.pdf

## Figures and Tables

**Table 1:** Differentially expressed genes associated with higher NIHSS scores at different time points post-stroke (PS)

Gene	Function	Subtype	Time PS	p-value		r
TSPO	Anti-inflammatory	all	24 h	0.004	↓	−0.106
TSPO	Anti-inflammatory	classical	24 h	0.003	↓	−0.095
TLR8	Pro-inflammatory	Non-classical	24 h	0.032	↑	0.060
CD86	Pro-inflammatory	all	3–5 days	0.010	↓	−0.089
IL-1β	Pro-inflammatory	all	3–5 days	0.018	↓	−0.105
CD33	Anti-inflammatory	intermediate	3–5 days	0.027	↓	−0.064
CD40	Pro-inflammatory	Non-classical	3–5 days	0.022	↓	−0.083
CCR2	Monocyte infiltration	all	3 months	0.002	↑	0.360
SLC24A4	cellular calcium ion homeostasis	all	3 months	0.049	↓	−0.255
STAT6	Anti-inflammatory	all	3 months	0.021	↑	0.306
CCR2	Monocyte infiltration	classical	3 months	0.010	↑	0.334
CD91	Immune modulator	classical	3 months	0.049	↓	−0.372
SLC24A4	cellular calcium ion homeostasis	classical	3 months	0.044	↓	−0.199
CCR2	Monocyte infiltration	intermediate	3 months	0.049	↑	0.362
CD11b	Monocyte adhesion and migration	intermediate	3 months	0.022	↑	0.266
CD91	Immune modulator	Non-classical	3 months	0.031	↓	−0.478
IL-10	Anti-inflammatory	Non-classical	3 months	0.035	↓	−0.376
SLC24A4	cellular calcium ion homeostasis	Non-classical	3 months	0.006	↓	−0.509

This table presents genes whose expression levels in primary human monocytes and their subtypedetermined by PCR significantly correlated with NIHSS scores, based on Pearson correlation analysis (P-value < 0.05). An upward arrow (↑) denotes increased gene expression, while a downward arrow (↓)indicates decreased expression with increasing NIHSS score.

## Data Availability

The R script and raw data files used in this study will be publicly available on GitHub at https://github.com/JTampe/Monocytes. This repository includes all necessary data and code to reproduce the results presented in this study. Users will be able freely access, download, and use the materials under the terms specified in the repository. For any additional inquiries, please contact the repository maintainer via the contact information provided on the GitHub page.
